# Preoperative diagnosis and staging of rectal cancer using diffusion-weighted and water imaging combined with dynamic contrast-enhanced scanning

**DOI:** 10.3892/ol.2014.2590

**Published:** 2014-10-09

**Authors:** QILI ZHAO, LIJIAN LIU, QIUYAN WANG, ZEXIA LIANG, GAOFENG SHI

**Affiliations:** 1 Department of Magnetic Resonance and Computed Tomography, The People’s Hospital of Langfang City, Langfang, Hebei 065000, P.R. China; 2 Department of Magnetic Resonance and Computed Tomography, The Fourth Hospital of Hebei Medical University, Shijiazhuang, Hebei 050019, P.R. China

**Keywords:** magnetic resonance imaging, rectum, rectal cancer, staging

## Abstract

The aim of the present study was to evaluate the value of diffusion-weighted imaging (DWI) and water imaging combined with dynamic contrast-enhanced scanning for the preoperative diagnosis and staging of rectal cancer. In total, 72 patients with pathologically confirmed rectal cancer were selected for examination using magnetic resonance imaging (MRI) with phased-array coils, DWI, water imaging and dynamic contrast-enhanced scanning. The patients were divided into two groups, experimental (simple enhanced scanning plus diffusion combined with water imaging) and control (simple enhanced scanning), for the pathological observations. The sensitivity, specificity and accuracy for the T staging of the carcinomas using scan enhancement with DWI and the evaluation of cancer using water imaging were 98.5% (65/66), 66.7% (4/6) and 95.8% (69/72), respectively, and the accuracy for N staging was 89%. Whereas, the sensitivity, specificity and accuracy for the T staging of the carcinomas using simple scan enhancement were 85.7% (42/49), 78.3% (18/23) and 83.3% (60/72), respectively, and the accuracy for N staging was 61%. Therefore, the combination of multiple MRI techniques may be of high value for the early diagnosis and exact staging of rectal cancer.

## Introduction

Rectal cancer is one of the most common malignancies of the digestive tract, ranking first in the worldwide incidence of malignant tumours. Furthermore, the incidence of rectal cancer in China shows an increasing trend, and individuals with the disease under the age of 40 years account for >30% (1). The five-year survival rate following radical surgery for rectal cancer is ~60%, with five-year survival rates of 80–90% for early rectal cancer (2). The early diagnosis, accurate staging and early treatment of rectal cancer are key to improving the five-year survival rate, particularly for the systematic screening of high-risk groups. Therefore, the early detection of rectal cancer is extremely important.

Magnetic resonance imaging (MRI) has the advantages of multidimensional imaging and clear display of the association between the rectum and its surrounding tissues, including the bladder, uterus and prostate. These advantages make MRI an important means of analysis for the preoperative evaluation of pelvic diseases. In addition, the use of MRI in staging rectal cancer has previously been reported (3,4), however, the majority of the studies have been performed using single screening technologies only. The simultaneous use of three types of cancer screening technologies, specifically enhanced, diffusion and water imaging, for the evaluation of local invasion and lymph node metastasis has not yet been reported. Therefore, the aim of the present study was to evaluate the efficiency of the combined application of three MRI examination techniques in the staging of advanced rectal cancer preoperatively. The results were compared with the surgical and pathological observations.

## Material and methods

### Clinical data

The clinical data of 72 patients (40 males and 32 females) with rectal cancer, aged between 17 and 79 years (mean age, 58.4 years), were collected between January 2008 and February 2012. The major patient clinical presentations included haematochezia, mucus bloody stool, changes in defecation habits, increasing stool frequency and abdominal pain. Following the MRI examinations, tumours were resected by local excision (tumours of stage T1 and partial stage T2), or total excision of the rectum and total mesorectal excision (tumours of stages T3 and T4 or patients with lymph node metastasis). This study was conducted in accordance with the declaration of Helsinki and approval was obtained from the Ethics Committee of the People’s Hospital of Langfang City (Langfang, China). Written informed consent was obtained from all participants.

### MRI examination methods

Phenolphthalein or magnesium sulphate was orally administered to the patients following food the evening prior to the MRI examination to clean the intestinal tract. For the MRI, 500–1,000 ml of warm water was infused into the rectum. Patients were placed in the supine position and the centre of the magnetic field was located on the iliac crest. The Avanto 1.5T MRI scanner purchased from Siemens AG (Berlin, Germany) was used for examination.

The diffusion-weighted imaging (DWI) technique with two b values (b=0 and 1,000) was used to measure the apparent diffusion coefficient (ADC) values of the normal intestinal and tumour tissues, as well as enlarged lymph nodes.

In addition, the water imaging techniques were performed using a turbo spin-echo (SE) sequence and the collected repetitive T2-weighted images were reconstructed using three-dimensional (3D) maximum intensity projection (MIP) to obtain 3D images.

Finally, enhanced scanning was performed using 3D volumetric interpolated breath-hold examination sequences following simple scanning. The acquisition matrix, field of view and slice thickness were the same as those used for simple scanning, specifically with a repetition time (TR) of 3.37 ms, echo time (TE) of 1.66 ms and Aver 1, respectively. Next, 15–20 ml of gadolinium-dimeglumine was used as the contrast agent through antecubital intravenous injection using a high-pressure syringe with a flow rate of 3 ml/sec. MRI scans were immediately performed following bolus with axial imaging, complemented by sagittal and coronal images.

### Specimen processing

The specimens were fixed with formaldehyde for 24 h and successive cross sections (5 mm thick) were performed and staged in accordance with the pathological criteria for tumour node metastasis (TNM) staging (5).

### Image analysis

All the MRI image data of the patients were divided into two groups of 72 patients each. One group (the control group) was analysed only with simple enhanced images and conventional T1-weighted imaging (T1WI) and T2-weighted imaging (T2WI) sequences; whereas the other group (the experimental group) was analysed based on enhanced images, DWI images and water images with conventional T1WI and T2WI sequences. The invasion of the rectal wall layers and surrounding organs, as well as pelvic lymph node size were observed. Lymph nodes of >5 mm in diameter, or iliac blood vessels along the distribution of lymph nodes with diameters of >1 cm were used as a diagnostic standard of lymph node metastasis. Accurate staging was performed according to the TNM staging method of the Union for International Cancer Control to guide the determination of clinical treatment programmes (5).

### Evaluation indices

The normal structure of the intestinal walls using MRI can be divided into three layers (6). On T2WI, the high signals in the innermost walls present the mucosa and submucosa (these signals were not distinguished in the current study), while the moderately low signals present the muscularis propria. Furthermore, the high signals in the outer intestinal walls present the fat surrounding the intestines. These layers were all surrounded by line-like low signal structures, which present the mesorectal fascia.

The criteria for the diagnosis of rectal cancer using MRI were determined according to Brown *et al* (7). The intestine demonstrated irregular filling defect and irregular valve walls. In this study the the lesions in the distal end showed ‘cuff’ or ‘truncated’ characteristics on MR water imaging (8). The diagnostic criteria of DWI (9) were the high signal intensity of the rectal walls and tumours, or rectal wall thickening in conventional sequences.

The current diagnostic criteria for T staging using MRI was recommended by the Union for International Cancer Control (UICC) and the American Joint Committee on Cancer (10). Additionally, the diagnostic criteria for N staging using MRI were determined according to Brown *et al* (11). Furthermore, the staging criteria developed by the UICC in 2009 was used as the pathological staging criteria for TN (5).

### Statistical analysis

SSPS 11.0 statistical software (SPSS, Inc., Chicago, IL, USA) was used for statistical analysis and the sensitivity, specificity and accuracy of rectal cancer stages were analysed by the simple scanning inspection method for MRI. Combining the sensitivity, specificity and accuracy of DWI and water imaging ultimately verified the diagnostic values of the various methods. P<0.05 was considered to indicate a statistically significant difference.

## Results

### Pathological typing and location

**Figure 1:**
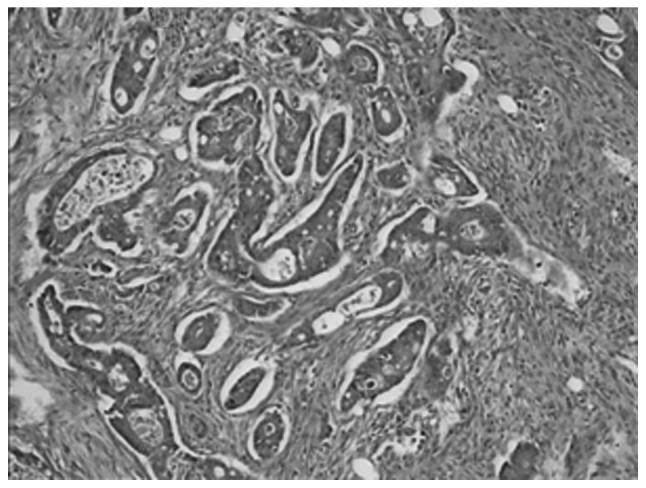
Pathological images showed that the whole intestinal wall layer had been invaded by moderately differentiated adenocarcinoma, involving the surrounding tissue lymph nodes (2/10) (stain, haematoxylin and eosin; magnification, 10×10).

The postoperative pathology for the 72 cases of rectal cancer were as follows: 61 cases of adenocarcinoma [five highly differentiated, 41 moderately differentiated (Fig. 1), 13 moderately and poorly differentiated and two poorly differentiated adenocarcinomas]; seven cases of mucous gland cancer; two cases of carcinoid; one case of adenosquamous carcinoma; and one case of malignant melanoma. Of these cases, 35 cases had tumours located in the upper rectum (10–15 cm from the anal margin), 26 cases had tumours in the rectal midpiece (5–10 cm from anal margin) and 11 cases had tumours in the lower segment of the rectum (<5 cm from the anal margin).

### Pathological T staging

Overall, one case was determined as stage T1, 43 cases were determined as stage T2, 26 cases were determined as stage T3 and two cases were determined as stage T4. In addition, a total of 543 lymph nodes were found in 62 cases, including 427 benign lymph nodes in 60 cases (34 stage N0 cases and 26 cases with mixed benign and malignant lymph nodes). Malignant lymph nodes were present in 116 cases (28 cases with mixed benign and malignant lymph nodes and two cases with malignant lymph nodes, including 18 stage N1 and 12 stage N2 cases) (Fig. 1).

The mean ADC value of the tumour tissues was 1.1076±0.3289×10^−3^ mm^2^/sec, whereas the average ADC value of the normal intestinal walls was 1.7056±0.2217×10^−3^ mm^2^/sec.

### T staging results of MRI examination

**Figure 2:**
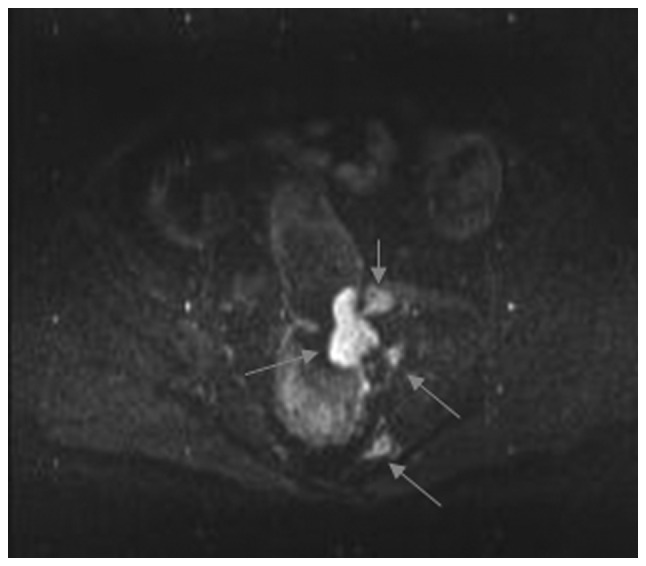
Diffusion-weighted imaging with b=1,000 showed clear, high signals in the tumours and three lymph nodes, exhibiting one or two more lymph nodes than those identified by simple enhanced scanning as indicated by the arrows.

**Figure 3:**
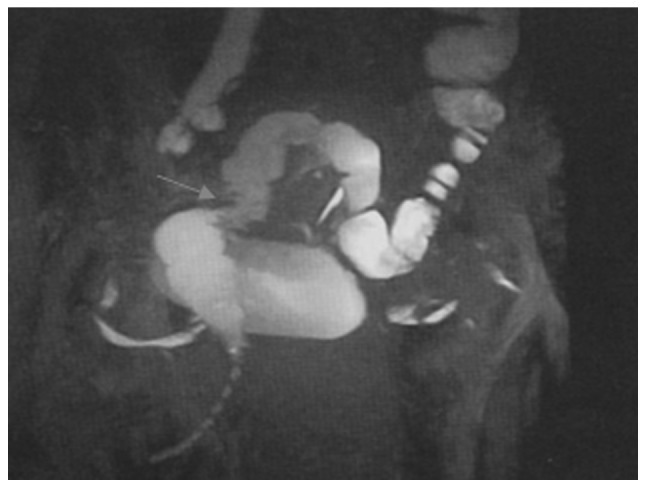
Irregular filling defect was identified in the intestinal cavity by water imaging.

The sequences of the simple enhanced scanning plus diffusion combined with water imaging for the the T staging of the experimental group were as follows: Overall sensitivity of 98.5% (65/66), total specificity of 66.7% (4/6) and overall accuracy of 95.8% (69/72). Two cases were overestimated and three cases were underestimated, while one stage T1 patient was correctly staged. The observation of a small tumour (~1 cm), with pathological invasion to the shallow muscle layer resulted in the underestimation of one stage T2 case as stage T1. In stage T2, the sensitivity was 94.7% (36/38), specificity was 80% (4/5) and accuracy was 93% (40/43). line strips of signal shadow in the lesion edge, uneven signals from the surrounding fat-free areas (mixed signals including the equisignal and the high-phase signal) and the observation of small nodules was the result of two stage T2 cases being overestimated as stage T3. In stage T3 (Figs. 2 and 3), the sensitivity was 94.7% (36/38), specificity was 75% (3/4) and accuracy was 92.3% (24/26). Two stage T3 cases were underestimated to be stage T2. Finally, in stage T4, the sensitivity, specificity and accuracy were all 100% (2/2).

**Figure 4:**
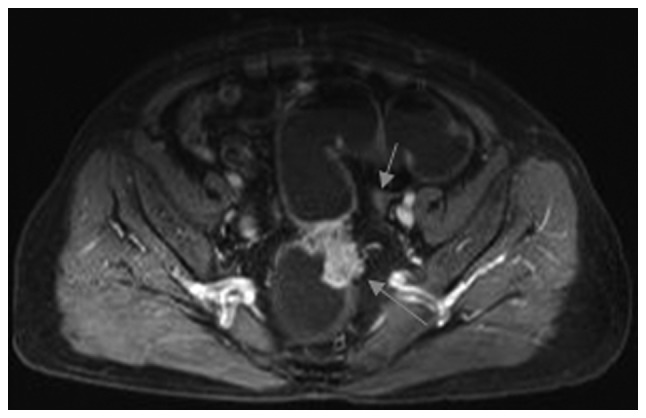
In the arterial stage, marked enhancement of the tumour and lymph nodes, as well as tumour invasion into the muscle layers, were observed.

**Figure 5:**
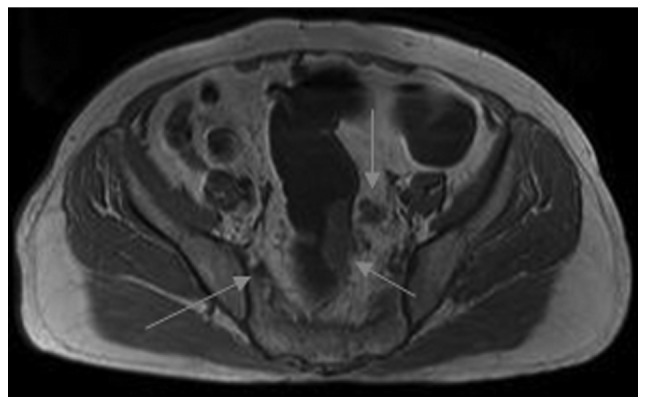
T1-weighted image without fat suppression showed tumour invasion of the surrounding fat tissue and two lymph nodes showed the same signal intensity at the left and right side of the intestinal walls with irregular shapes.

The sequences of the simple enhanced scanning for the T staging of the control group were as follows: Overall sensitivity of 85.7% (42/49), total specificity of 78.3% (18/23) and overall accuracy of 83.3% (60/72). In total, five cases were overestimated and seven cases were underestimated. In stage T1, two stage T2 patients were underestimated as stage T1. In stage T2, the sensitivity was 86.7% (26/30), specificity was 84.6% (11/13) and accuracy was 86% (37/43). Two stage T2 patients were underestimated to be stage T1, whereas four cases were overestimated as stage T3. In stage T3 (Figs. 4 and 5), the sensitivity was 78.6% (11/14), specificity was 75% (9/12) and accuracy was 76.9% (20/26). Three stage T2 patients were underestimated to be stage T2, whereas three cases were overestimated as stage T4. Finally, the sensitivity, specificity and accuracy of stage T4 were all 100% (2/2) (Tables I and II).

### N staging results of MRI examination

The sequences of the simple enhanced scanning plus diffusion combined with water imaging for the N staging of the experimental group were as follows. A total of 454 lymph nodes were found in 57 cases, including 346 benign lymph nodes (with equal DWI signals or of regular shapes) with an average ADC value of 0.9126±0.1945×1^−3^ mm^2^/sec, as well as 108 malignant lymph nodes (with high DWI signal or of irregular shapes) with an average ADC value of 0.7691±0.1193×10^−3^ mm^2^/sec. In total, 30/34 cases were determined as stage N, 16/18 cases were determined as stage N1 and 11/12 cases were determined as stage N2. The overall accuracy of the N staging was 89%.

The sequences of the simple enhanced scanning for the N staging of the control group are as follows. A total of 28/34 cases were determined as stage N0, 5/18 cases were determined as stage N1 and 6/12 cases were determined as stage N2. The total accuracy of the N staging was 61% (Table III).

## Discussion

The main treatment for rectal cancer is surgery, which cures the majority of early-stage patients. However, the majority of patients have no treatment options at diagnosis. With the development of novel treatment methods, such as preoperative radiotherapy, systemic or intra-arterial chemotherapy, surgical resection of liver metastasis, and preoperative radiotherapy and chemotherapy, the tumour size can be reduced and thereby improve the resection rate (12). The aim of the current study was to provide important references for formulating preoperative treatment plans using accurate imaging staging, particularly for patients with tumours that could not be directly surgically treated, but could be surgically resected following radiotherapy or chemotherapy.

The imaging examination of rectal cancers includes endorectal ultrasound, computed tomography (CT) and MRI. The accuracy of T staging for rectal cancer in the rectal cavity using endorectal ultrasound is 86%, and the accuracy of N staging is 62–83% (13). Rectal stenosis and upper rectum diseases are the major limitation factors of endorectal ultrasound due to inflammatory infiltration of the tumour edges. In addition, the tumour invasion is difficult to distinguish and the morphological changes of the outer edges of the muscular layers caused by muscular contraction may be misdiagnosed as tumours breaking through the muscular layers. The rectal tumours, surrounding invasion, distant metastasis and postoperative recurrence can be displayed on CT. However, CT is unable to accurately distinguish the intestinal wall layers and is insensitive to the slight infiltration of fat. Thus, preoperative T staging by CT has certain limitations and the determination of lymph node metastasis based on the size and shape of lymph nodes lacks specificity (13).

The resolution of MRI for soft tissue is high and axial, sagittal and coronal 3D imaging clearly shows the rectal mucosa, submucosa, muscularis and serosa of the intestine and tumours, as well as the surrounding fat tissues and organs. The accuracy of the T staging of rectal cancer using MRI is between 67 and 86%, whereas that for N staging is between 57 and 65% (14). In the present study, the accuracy of T staging using only conventionally enhanced sequences was 83.3%, whereas that for N staging was 61%. Furthermore, the accuracy of T staging using simple enhanced scanning plus diffusion combined with water imaging was 95.8%, and that for N staging was 89%, which was significantly improved. The results indicated that the combined application of various inspection technologies, such as simple enhanced scanning, DWI and water imaging, has important effects that contribute to the correct diagnosis of rectal cancer, significantly improving the accuracy of TN staging.

In the current study, irregular soft tissue lumps, localised or diffused intestinal wall thickening and irregular luminal stenosis in the rectal cavity were shown in the MRI scanning. In simple scanning, the 72 cases exhibited slightly low signals or equal signals on T1WI, and mixed low and high signals on T2WI. In addition, seven cases with mucinous adenocarcinoma exhibited significantly high signals on T2WI, which is consistent with the literature (15) and is the result of a mucus lake formed by the large amounts of mucus secreted by the tumours. Thus, the prognoses were poorer than those of the non-mucinous adenocarcinomas (15). Homogeneous or heterogeneous enhancements were shown in the tumours and the edges were smooth, nodular or jagged on enhanced scanning.

In rectal cancer staging, the involvement of the circular muscle is key to identifying stages T1 and T2, and the invasion of the perirectal fat tissues is key to identifying stages T2 and T3. Furthermore, the normal rectal circular muscle layers show circular homogeneous low signals on T2WI. This complete ring indicates that the cancer has not broken through the myometrium, which is considered to indicate stages T1 and T2. In addition, no involvement of the annular muscle is considered to indicate stage T1, whereas the involvement of the circular muscle that does not exceed its outer edges and surrounding fat tissue are considered to indicate stage T2 (16). However, the identification of lesions of stages T1 and T2 using MRI is limited, as observed in the two groups of the current study with the underestimation of stage T2 as stage T1. This observation indicated that the differentiation between stages T1 and T2 using DWI and water imaging has no evident significance. The invasion of the serosa and surrounding fat tissues is the basis for identifying stages T2 and T3 and is criteria for performing neoadjuvant therapy (17). Invasion of the muscle layer, as well as to the surrounding nodules or lumps by the tumour tissues are considered to indicate stage T3. Furthermore, the adipose tissues surrounding the intestine are shown well without fat suppression on T1WI and T2WI sequences, which is beneficial for identifying stages T2 and T3.

For DWI sequences, one study (18) compared the image quality with the ADC values obtained with different b values and concluded that b=1,000 sec/mm^2^ is a reasonable b value for the DWI of colorectal cancer. Thus, the above parameters were used in the present study. Based on the value of b=1,000 sec/mm^2^, all tumour lesions showed high signals, with stark contrast to the intestinal walls, faeces with low signals and significantly increased diagnostic sensitivity (98.5%) and accuracy (95.8%). Quantitative measurement of the ADC value is an additional important tool for the analysis of benign and malignant tumours, as well as lymph nodes using DWI imaging, which reflects the diffusion properties of water molecules, with size depending on the imaging material and spatial distribution of the internal molecules (19). The ADC value of the surrounding normal intestinal tissues was significantly higher than that of the rectal tumour tissues, which is predominantly attributed to the high content of tumour cells in tissues, dense cell structures, close and disorder arrangement, free water reduction in the cell gaps, water diffusion limitations and decreasing ADC value (20). Therefore, the ADC value with different large b values may present an effective tool for rectal tumour diagnosis.

The evaluation of lymph nodes using MRI combined with enhanced scanning relies only on tumour size. Thus, the internal structures of the lymph nodes are not shown. Only using the tumour size as the standard for assessing metastasis has some limitations, such as the inability to identify whether enlarged lymph nodes are the result of inflammation, reactive lymph node hyperplasia or metastasis, and the failure to detect small but metastasized lymph nodes. The number of perirectal lymph nodes with diameters of >5 mm or distributed along the iliac vessels and groin with diameters of >1 cm detected by MRI scanning and enhanced scanning was less than that observed during surgery. It was not possible to individually compare the swollen lymph nodes shown on MRI with those observed during surgery, thus, the correlation between lymph node metastasis and size could not be accurately determined. Therefore, N staging by MR scanning combined with enhanced MRI scanning exhibited more evident limitations. The water diffusion of the metastasized cancer lesions or the internal structure of lymph nodes was restricted, similar to that of the primary tumour. The same high signals were displayed using DWI with or without irregular margin changes, and the two ADC values were significantly reduced. Thus, the missing or unclearly displayed cancer lesions and lymph nodes in the conventional sequences of the simple scanning combined with enhanced scanning can be observed on DWI-weighted imaging, which significantly improves the sensitivity and accuracy of N staging. A previous study (21) reported that the evaluation of lymph node metastasis for rectal cancer using DWI sequences was significant and that the differentiation between benign and malignant lymph nodes may be determined through the ADC value of the lymph nodes. The study further confirmed this hypothesis and the accuracy of N staging was improved from 61 to 89%.

On MRI combined with water scanning, the appearence of the rectum is a hollow organ with a specific anatomical location. MR water imaging was used with a long TR (>3,000 ms) and extremely long TE (>150 ms) to obtain the repeat T2WI effect, as well as to image the excretory organs. The rectum was filled with water prior to examination and significant signal reduction was observed in the lesions of the internal rectal cavity or thickened intestinal walls, as well as the surrounding structures. The signals of the aqueous rectal cavity were clearly displayed and the narrow characteristics and extent of the lesion in the intestinal cavity were clearly displayed through 3D or 2D MIP reconstruction. The scope of the surgical resection and the distance of the lesion from the anus can also be visually displayed. Compared with barium enema, it is possible to display tumour invasion of the proximal segment for the intestinal obstruction and to identify invasion of the tissues and organs outside the intestinal cavity by MRI (15,16).

However, DWI and water imaging have poorer qualities than the SE sequences and the spatial resolution is lower. Thus, TN staging for tumours using only DWI and water imaging is infeasible. However, the combined application of dynamic contrast-enhanced MRI, DWI and water imaging result in more accurate images for the TN staging of tumours than those obtained by enhanced MRI only (22). In the current study, the location of the tumours determined using the combined application of DWI and water imaging was clearer and the dynamic contrast-enhanced MRI images compensated for the lower image resolution of the DWI and water imaging.

MRI can be used with multiple parameters and sequences for good resolution of soft tissues and a wide scan range without space restrictions. In addition, the thickness of the intestinal walls, anatomical structures and neighbouring structures are clearly shown. Lumps in the intestinal wall and their size, as well as adjacent organ invasion can be exhibited at cancer diagnosis. Furthermore, lymph node swelling and abdominopelvic cavity metastasis may be observed (23). In the current study, the signal changes of the MRI were quite specific, which has high clinical value in disease diagnosis, staging, assessment of resectability and other aspects. The sensitivity and accuracy of the diagnosis for elevated lesions of >10 mm in size were all >95%, and the ADC values provided a reasonable basis for the identification of benign and malignant rectal lesions and aid in the selection of a suitable chemotherapy and chemoradiotherapy.

Limitations of the current study included the following: i) The positive lymph nodes detected by imaging did not correspond to those observed during surgery, which has a certain influence on the evaluation of the positive lymph nodes; and ii) the capabilities identified for stage T1 and T2 lesions were limited, and patients of stage T2 were underestimated as stage T1 in the two groups. This observation indicated that no method is superior to the other in terms of the differentiation between stages T1 and T2.

In conclusion, the multi-sequencing combined application of MRI is important for the early diagnosis and accurate staging of rectal cancer, which may improve the formulation of clinical treatment plans. In addition, DWI imaging is important for improving the sensitivity of T staging and contributes to the enhanced accuracy of N staging combined with the determination of the ADC value. However, the application of this method alone is not suitable for cancer diagnosis and staging. In addition, the application of water imaging alone, which is important for clearing the range of tumour imaging, is also not recommended for cancer diagnosis and staging.

## Figures and Tables

**Table I:** MRI and pathological T staging results for rectal cancer.

	Experimental MRI group, n				Control MRI group, n			
		
Pathological T staging	T1	T2	T3	T4	T1	T2	T3	T4
T1	1	0	0	0	1	0	0	0
T2	1	40	2	0	2	33	8	0
T3	0	2	24	0	0	10	16	0
T4	0	0	0	2	0	0	0	2
Total	2	42	26	2	3	43	24	2
Accuracy (%)	50	88.9^a^	85.7^a^	100	33.3	62.3	47	100

χ^2^ test; t value=2.675; P=0.011.

a P<0.05 vs. control group was considered to indicate a statistically significant difference.

MRI, magnetic resonance imaging.

**Table II:** Statistical analysis of the T staging results of the two groups.

Groups	Sensitivity, %	Specificity, %	Accuracy, %
Experimental^a^	98.5	66.7	95.8
Control^b^	85.7	78.3	83.3
P-value	<0.05	<0.05	<0.05

a Simple enhanced scanning plus diffusion combined with water imaging; and

b simple enhanced scanning. Statistical analysis was performed by χ^2^ test.

**Table III:** Lymph node status of MRI and pathological examination.

	Experimental MRI group for, n			Control MRI group, n		
		
Pathological N staging	N_0_	N1	N2	N_0_	N1	N2
N0	30	1	0	28	9	4
N1	2	16	1	5	5	6
N2	2	1	11	1	4	2
Total	34	18	12	34	18	12
Accuracy (%)	85.7	76.2^a^	92.3^a^	58.3	17.2	12.5

Statistical analysis was performed by χ^2^ test. P<0.05 was considered to indicate a statistically significant difference.

a P<0.01 vs. control group.

MRI, magnetic resonance imaging.
